# Effect of maternal periconceptional undernutrition in sheep on cortisol regulation in offspring from mid-late gestation, through to adulthood

**DOI:** 10.3389/fendo.2023.1122432

**Published:** 2023-02-02

**Authors:** Mark H. Oliver, Anne L. Jaquiery, Kristin L. Connor, Hui Hui Phua, Jane E. Harding, Eric B. Thorstensen, Frank H. Bloomfield

**Affiliations:** ^1^ Liggins Institute, University of Auckland, Auckland, New Zealand; ^2^ Department of Health Sciences, Carleton University, Ottawa, ON, Canada

**Keywords:** programming, long-term, outcomes, adrenal, cortisol

## Abstract

**Introduction:**

Maternal periconceptional undernutrition (PCUN) alters fetal hypothalamic-pituitary-adrenal axis (HPAA) function and placental glucocorticoid metabolism in sheep. The effects of PCUN on HPAA function in adult life are not known. We investigated the effects of PCUN on fetal adrenal development across gestation and on cortisol regulation in adult offspring.

**Methods:**

Ewes were undernourished from 61 days before to 30 days after conception (‘PCUN’) or fed *ad libitum* (‘N’). mRNA expression in the fetal adrenal gland of ACTH receptor (*ACTHR*), steroidogenic acute regulatory protein (*STAR)*, cytochrome P450 17A1 (*CYP17A1)*, 11beta-hydroxysteroid-dehydrogenase type 2 (*11βHSD2)*, insulin-like growth factor-2 (*IGF2)*, and in the fetal hippocampus of *11βHSD1*, *11βHSD2*, mineralocorticoid receptor (*MR*) and glucocorticoid receptor (*GR*) was determined at 50 (adrenal only), 85, 120 and 131 days of gestation (term=148 days). In adult offspring (≥ 3 years, N; 10 female, 5 male, PCUN; 10 female, 10 male) a combined arginine vasopressin (AVP, 0.1 μg/kg) and corticotropin-releasing hormone (CRH, 0.5 μg/kg) challenge and a metyrapone (40 mg/kg) challenge were undertaken. mRNA expression of *ACTHR*, *STAR* and *CYP17A1* were determined in adult adrenals.

**Results:**

Fetal adrenal *STAR*, *CYP17A1* and *IGF2* mRNA expression were not different between groups in early gestation but were higher in PCUN than N at 131 days’ gestation (all *p*<0.01). PCUN reduced fetal hippocampal *MR* and *GR* mRNA expression by 50% at 85 day, but not in later gestation. Adult offspring plasma cortisol responses to AVP+CRH or metyrapone were not different between groups. Plasma ACTH response to AVP+CRH was lower in PCUN males but ACTH response to metyrapone was not different between groups. Adult adrenal *ACTHR*, *STAR*, and *CYP17A1* mRNA expression were not affected by PCUN.

**Conclusions:**

We conclude that the effects of PCUN on fetal HPAA function that became apparent in late gestation, are not reflected in adrenal cortisol secretion in mid-adulthood.

## 1 Introduction

Poor maternal nutrition around the time of conception is associated with an increased risk of impaired glucose regulation, cardiovascular disease and obesity in the offspring (1). Sheep studies indicate that the effects of periconceptional maternal undernutrition (PCUN) may be mediated by altered maternal and fetal hypothalamo-pituitary-adrenal axis (HPAA) function (2, 3) and placental glucocorticoid metabolism (3–5) during pregnancy. Central to this phenomenon is the concept that in the face of an adverse maternal environment, adaptations made for maintenance of pregnancy and survival of offspring from birth to reproductive age may lead to a mismatch with later environmental influences during postnatal life (6). Longitudinal studies in large mammals, such as the sheep, have the potential to investigate potential mechanisms as well as effects of the exposure through to adulthood within a reasonable timeframe.

The periconceptional period is generally defined as that extending from oocyte maturation, follicular development, conception, and the embryonic blastocyst stage up until implantation (7). Maternal nutrition around this time may have powerful effects on hormonal axes such as the HPAA (4). The effects of PCUN may be found in tissues and organs of different germ line origin such as muscle (8), gonads (9) and regions of the brain such as the hypothalamus, and likely are mediated by epigenetic modifications of DNA (10), such as methylation (11).

Precocious activation of the fetal HPAA in response to PCUN has been reported in sheep (12); however, in the same paradigm, by young adulthood circulating plasma cortisol responses to AVP+CRH stimulation were depressed (13). The level of the HPAA at which this suppression of cortisol responses is regulated is not known, nor is it clear whether the postnatal suppression of cortisol response in PCUN offspring persists to later adulthood. Alterations on HPAA responsiveness may have very important implications for risk of disease in later life, for example *via* immune function (14, 15).

We aimed to characterize the effects of PCUN on the ontogeny of fetal HPAA development and on HPAA function in mid-adulthood, in response to stimulation by both corticotropic hormones and blockade of cortisol synthesis.

## 2 Materials and methods

### 2.1 Animals

All animal experimentation was approved by the University of Auckland Animal Ethics Committee (Approvals AEC/07/2004/R277, AEC/07/2005/R389, and AEC/02/2008/R628) and conducted in accordance with those approvals.

Multiparous 4- to 5-year-old Romney ewes were acclimatized to a custom-made total concentrate ration (Dunstan Nutrition, Hamilton, NZ) over 10 days on pasture prior to feedlot entry (16). Ewes were then randomly allocated to 2 treatment groups: controls (N; daily feed intake 3-4% of body weight) or undernutrition from 61 days before to 30 days after mating (PCUN). Undernutrition involved a 2-day fast then individual adjustment of feed to achieve and maintain 10% body weight reduction (16). In undernourished ewes, feed intake after fasting was 1-2% of body weight per day but increased to around 80% of controls before restriction was lifted. Representative maternal weight data during pregnancy are reported elsewhere (17).

Ewes were housed indoors in a photoperiod-controlled feedlot from 70 days before mating until 2 weeks after lambing. While undergoing nutritional restriction ewes were housed individually in 1.2 × 1.4 m pens with mesh sides to allow visual contact between ewes. All ewes were housed in individual pens from 2 weeks before, until 2 weeks after lambing (term gestation is ~148 days after mating).

Two weeks before mating with Dorset rams, estrus was synchronized using an intravaginal progesterone release device (Eazi-Breed CIDR; Zoetis, NZ, Ltd) (18). Ewes identified as carrying singleton lambs by ultrasonography 50 days after mating were retained for the studies. Separate cohorts of singleton-bearing ewes were euthanized at 50 (N=7, PCUN=3), 85 (N=7, PCUN=8), 120 (N=12, PCUN=10) and 131 (N=9, PCUN=11) days of pregnancy for collection of fetal adrenal and hippocampal (except day 50) samples that were stored at -85°C for later mRNA analysis.

Ewes and lambs for the postnatal studies were returned to pasture 2 weeks after birth. Weight and size measurements been reported previously (19, 20). Lambs were weaned at 12 weeks of age. Between 3 and 4 years of age offspring (N: 10 females, 5 males; PCUN: 10 females, 10 males) were brought indoors and acclimatized for a week to full ration feeding with the same concentrate feed used in the maternal manipulations (~3% of body weight per day). The procedures were the same as those described for HPAA tests undertaken at 10 months, in the same animals (13). Indwelling catheters (size 040, Critchley Electrical, Auburn, Australia) were inserted into both jugular veins under local anesthesia. Ewes were fitted with CIDR releasing devices to prevent estrus (18) occurring during endocrine testing. Two to 3 days after catheter insertion animals underwent AVP + CRH challenge. After a 3 mL baseline blood sample, equimolar doses of bovine CRH (0.5 μg/Kg) and AVP (0.1 μg/kg, both Sigma Chemical Co., St Louis, MO, USA) were given intravenously. Further 3 mL blood samples were taken 15, 30, 45, 60, 120 and 240 minutes after the dose. Two days later blockade of cortisol synthesis was induced using Metyrapone (40 mg/Kg intravenously), supplied by Novartis, Basel, Switzerland, 09-MTA-046). Three mL blood samples were taken before blockade and then 30, 60 and 120 minutes later. Samples were centrifuged at 3,000 g and plasma split into two aliquots and stored at -20°C until analysis. Three days after the conclusion of the endocrine studies sheep were euthanized by anesthetic overdose (20). Adrenal tissues were snap-frozen in liquid nitrogen and stored at -85°C until later analysis.

### 2.2 Hormonal analysis

Plasma ACTH was measured using a commercial radioimmunoassay kit (DiaSorin Inc., Stillwater, MN, USA) previously validated for sheep (21); inter- and intra-assay coefficients of variation were 13.0% and 7.6%, respectively. Plasma cortisol and its precursor 11-deoxycortisol were measured by liquid chromatography tandem mass-spectrometry as described previously (22) with mean inter- and intra-assay coefficients of variation of 11.0 and 4.3%, respectively.

### 2.3 RNA isolation and cDNA synthesis

Adult adrenal was pulverized into powder using 6870 Freezer Mill Cryogenic Grinder (SPEX SamplePrep, USA). Fetal hippocampus and fetal adrenal were ground into powder using mortar and pestle in liquid nitrogen. Total RNA was extracted using TRIZOL (Invitrogen, USA). RNA concentration was measured and the absorbance ratios 260/280 and 260/230 with ratios ≥1.9 considered acceptable purity. RNA gels were run to check the RNA integrity. Total RNA (2.5 µg) was treated with RNase-free DNase I (Invitrogen, USA) before reverse transcriptase polymerase chain reaction (RT-PCR) to eliminate potential genomic DNA. First-strand cDNA was synthesized using SuperScript® *VILO* **™** *cDNA Synthesis* system (Invitrogen, USA).

### 2.4 Primers and Taqman probes

All primers and Taqman probes were designed using Primer Express Software (Applied Biosystems, USA). The following genes were studied, ACTH receptor (*ACTHR*), steroidogenic acute regulatory protein (*STAR)*, cytochrome P450 17A1 (*CYP17A1)*, 11beta-hydroxysteroid-dehydrogenase type 2 (*11βHSD2)*, insulin-like growth factor-2 (*IGF2)*, and in the fetal hippocampus *11βHSD1*, *11βHSD2*, mineralocorticoid receptor (*MR*) and glucocorticoid receptor (*GR*). A BLAST search ensured that primers and probes were not designed from homologous regions that would encode for genes other than our target genes (**Table 1**). Reference genes primer and probe sequences have been previously published (23).

**Table 1:** Primer and probe sequences for target genes.

Gene and GenBank Accession ID	Forward (5’ → 3’)	Reverse Forward (5’ → 3’)	Probe
**CYP17A1** Accession ID: L40335	TTCACCAGCGACTCCATCACT	TGTTGTTATTGTCTGCATTCACCTT	6FAM- ACTTGCTGCACATACT -MGBNFQ
**ACTHR** Accession ID: AF116874	TTGGAAAATGTTCTGATCATGTTCA	CATCTGCTGTGCTTTCAAAACTG	6FAM- TGGGTTACCTCGAGCCT -MGBNFQ
**STAR** Accession ID: AF290202	AAGGTCCTGCAGAAGATTGGAA	CGCCTCTGCAGCCAACTC	6FAM- AGACACGATCATCACTCA -MGBNFQ
**GR** Accession ID: S44554	GGGCCAACATAATTGGCAATAA	CCCAGAGGTACTCACACCATGA	6FAM- ATGTCTGCCATTTCT -MGBNFQ
**MR** Accession ID: AF349768	AAGGTGGAATCCGGGAATG’	GACGTCTGCGGTCACTTCCT	6FAM- CCAAACCCTTTACTTTC -MGBNFQ
**11β-HSD1** Accession ID: NM_001009395	CAGTTGCTGGGATATACAATGCA	CTTTGATAATCTCCAGGGCACAT	6FAM- AAGCATCTCCAAAGGA -MGBNFQ
**11β-HSD2** Accession ID: NM_001009460	TTCCGCACCTGCATGGA	AGTGGCAAGAGGCCTTTGG	VIC- ACTTCTTTGGTGCACTAGA -MGBNFQ
**IGF2** Accession ID: M89788	CGAGGCATCCAGCGATTAG	TAGATGGTGTCACTTGGCAGAATT	6FAM- AGTGAGCCAAAGTGTC -MGBNFQ

FAM/VIC, fluorescent reporter dyes bound to the TaqMan probe. MGBNFQ, molecular-groove binding non-fluorescence quencher.

### 2.5 Analysis and selection of stably expressed reference genes

For fetuses, real time PCR data were normalized with 18s; these studies were performed shortly after tissue collection to avoid any tissue deterioration from prolonged storage, and this was the method of normalization used at that time. For adult animals, analysis was ~4 years later and real-time PCR data were normalized with multiple stably expressed reference genes reflecting development in analytical approach over time. Using BestKeeper (24), *RPL19*, *GAPDH* and *HPRT1* were found to be the most stably expressed HKGs (with SD<1) amongst our seven candidate reference genes: *RPL19*, *GAPDH*, *HPRT1*, *YWHAZ*, *ACTB*, *CYPA*, *18S*.

### 2.6 Real time PCR and relative gene expression analysis

Real time PCR was carried out using ABI 7900HT Real-Time PCR system (Applied Biosystems). Samples were performed in triplicate and an average quantification cycle ( $\bar{Cq}$ ) value was obtained for each triplicate. Standard curves for the target genes and reference genes were included in the runs to calculate the amplification efficiencies (E) of the genes using E = 10^(-1/slope)^ (24) value was obtained for each triplicate. Standard curves for the target genes and reference genes were included in the runs to calculate the amplification efficiencies (E) of the genes using E = 10^(-1/slope)^ (24). The relative expression value (RE) in fetal tissue was calculated using mathematical model described by Pfaffl et al (25); the RE in adult tissue was calculated using a mathematical model that accounts for normalization against multiple reference genes (26, 27).

### 2.7 Statistical analysis

Basal hormonal and area under the curve (AUC) responses to challenge tests were analyzed initially by factorial ANOVA and then multiple regression with treatment group and sex as covariates (JMP software, version 15). Changes over time during the challenge tests were analyzed by repeated measures MANOVA with Tukey-Kramer correction for multiple comparisons. Significant differences were accepted at the 5% level. Data are presented as mean ± SEM. For mRNA, statistical analyses were conducted using R version 4.1.1. Gene expression results are presented in log_2_ fold change by log_2_ transformation of the RE value. Statistical differences in log_2_ fold change were analyzed using Student’s *t*-test. A *p* value < 0.05 was considered statistically significant.

**Figure 1:**
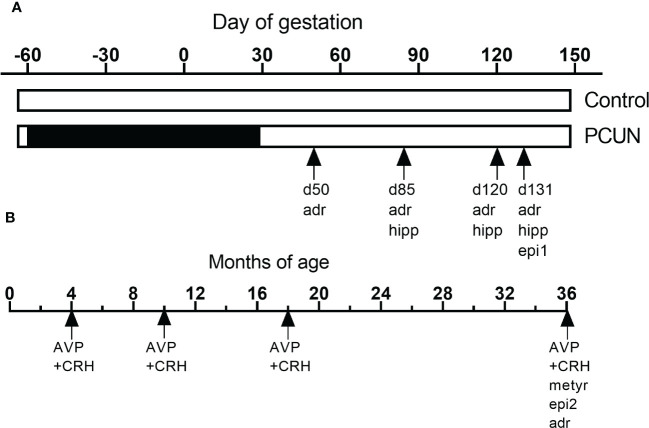
Schematic representation of the various studies using this cohort of animals during fetal **(A)** and postnatal life **(B)**. ‘adr’, mRNA expression in adrenals. ‘hip’, mRNA expression in hippocampus. ‘epi1’, fetal epigenetic studies (28). ‘epi2, epigenetic studies in adult (29). ‘‘AVP+CRH’, corticotropin stimulation tests in previous (13) and the current study. ‘met’, metyrapone blockade.

**Figure 1** shows how the current studies relate to other HPAA-related studies on this same cohort, previously undertaken (13, 28, 29)

## 3 Results

Growth and post-mortem morphometric data for both fetuses and adult offspring have been reported previously (17, 20).In brief, there were no effects of PCUN on body or adrenal weight in fetal life, at birth or in middle adulthood.

### 3.1 Fetal hippocampus

**Figure 2:**
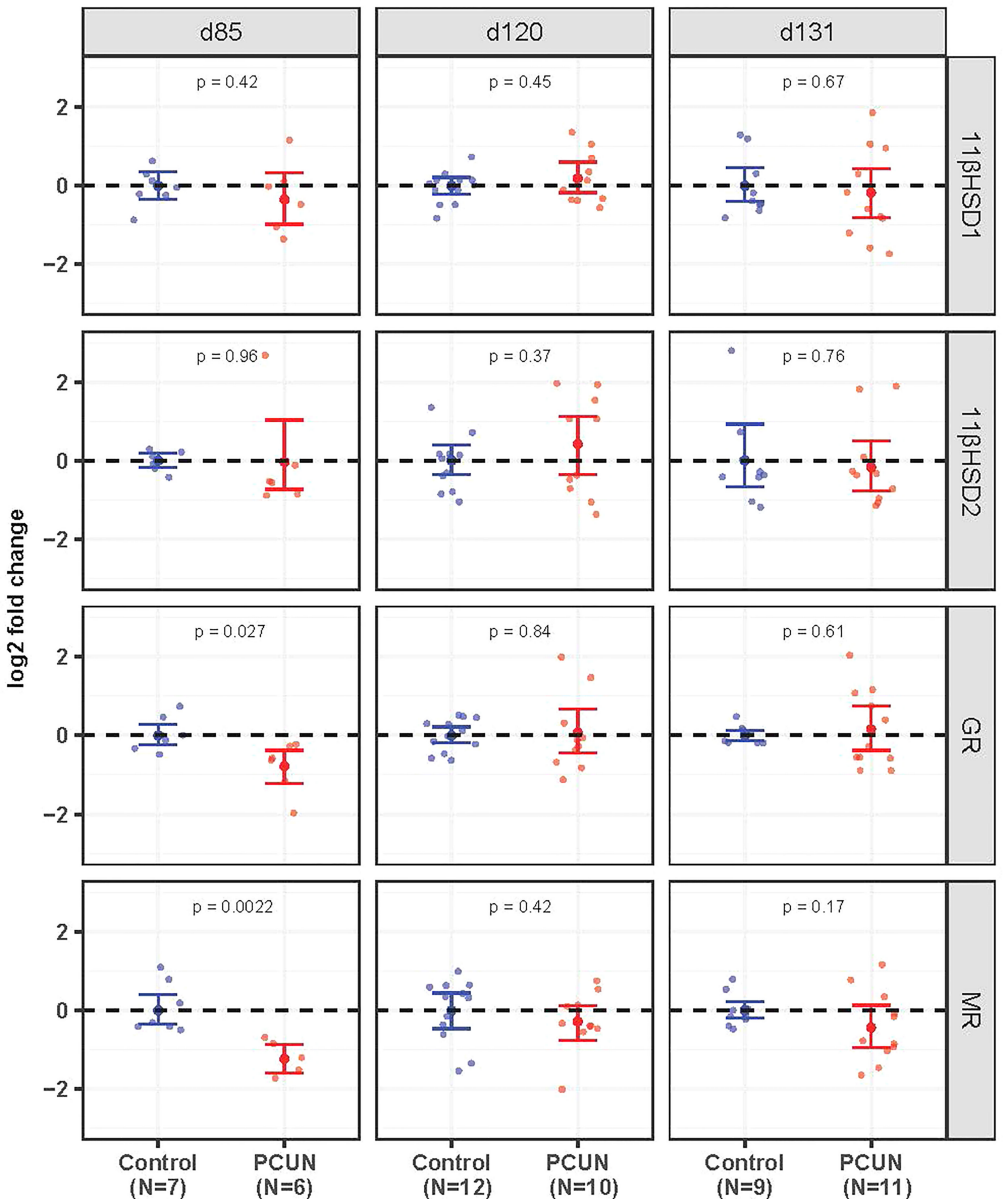
Fetal hippocampal mRNA expression. Log_2_ fold change of *11βHSD1*, *11βHSD2*, *GR* and *MR* mRNA expression levels in PCUN (symbol in red) in comparison to N (symbol in blue) based on real-time PCR analysis in the fetal hippocampus. Data are shown as mean ± 95% confidence interval. Symbols represent individual animals. Student’s t-tests were used to assess statistical difference, with significance defined as *p* < 0.05.

Hippocampal mRNA expression of both *GR* and *MR* mRNA was decreased in PCUN fetuses compared to their N counterparts at 85 days gestation (**Figure 2**, *p* < 0.05). There were no differences at the later ages examined. Expression of *11β-HSD1* and *11β-HSD2* in the fetal hippocampus were not affected by PCUN.

### 3.2 Fetal adrenal

**Figure 3:**
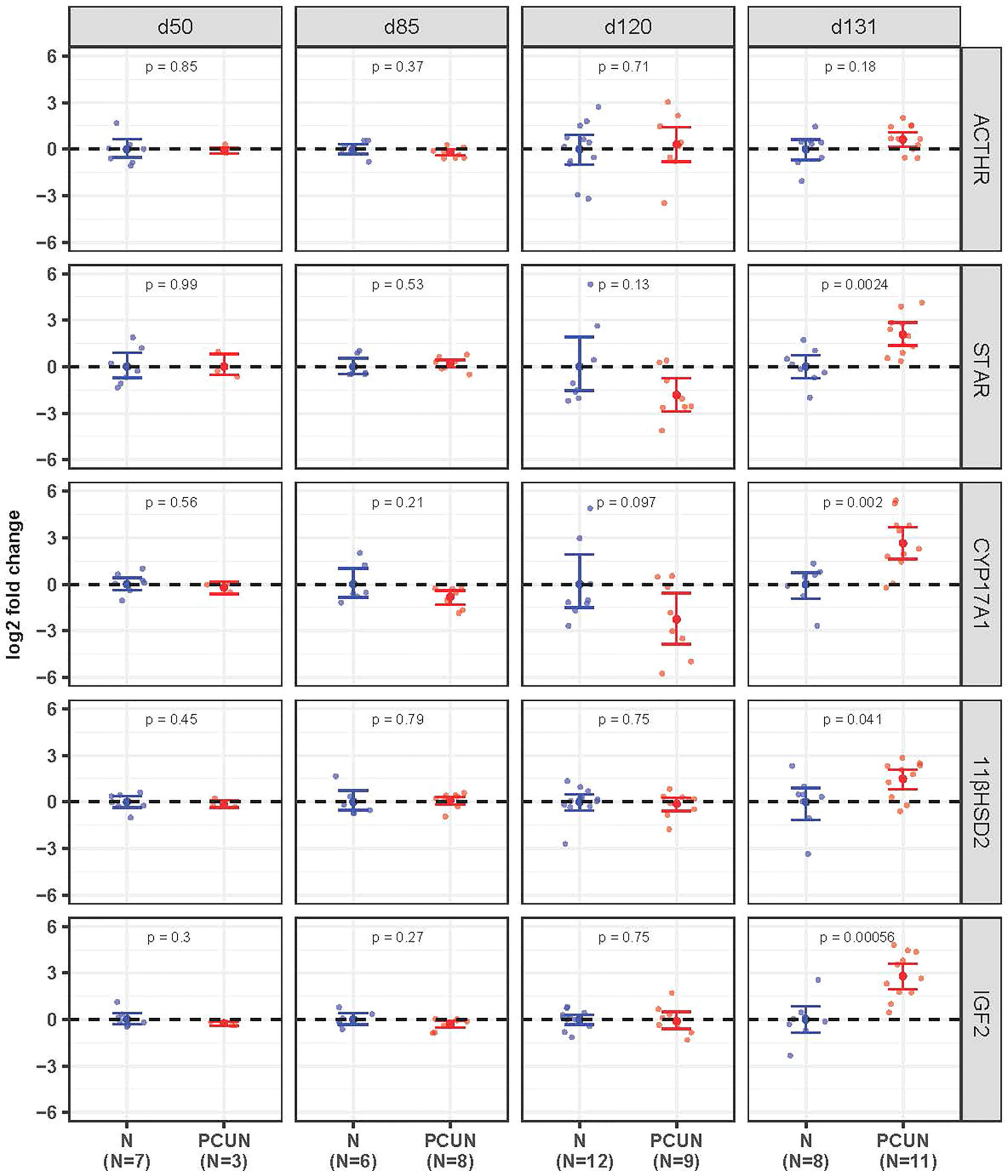
Fetal adrenal mRNA expression. Log_2_ fold change of *ACTHR*, *STAR*, *CYP17A1*, *11βHSD2*, and *IGF2* mRNA expression levels in PCUN (symbol in red) in comparison to N (symbol in blue) based on real-time PCR analysis in the fetal adrenal. Data are shown as mean ± 95% confidence interval. Symbols represent individual animals. Student’s t-tests were used to assess statistical difference, with significance defined as *p* < 0.05.

Prior to 131 days of gestation there were no effects of PCUN on expression of mRNA for any of the fetal adrenal targets examined (**Figure 3**). At 131 days expression of *STAR*, *CYP17A1*, *IGF2* and *11β-HSD2* mRNA were all higher in PCUN relative to N (all *p*<0.05), while *ACTHR* mRNA expression was unaffected.

### 3.3 Adult adrenal stimulation tests

**Figure 4:**
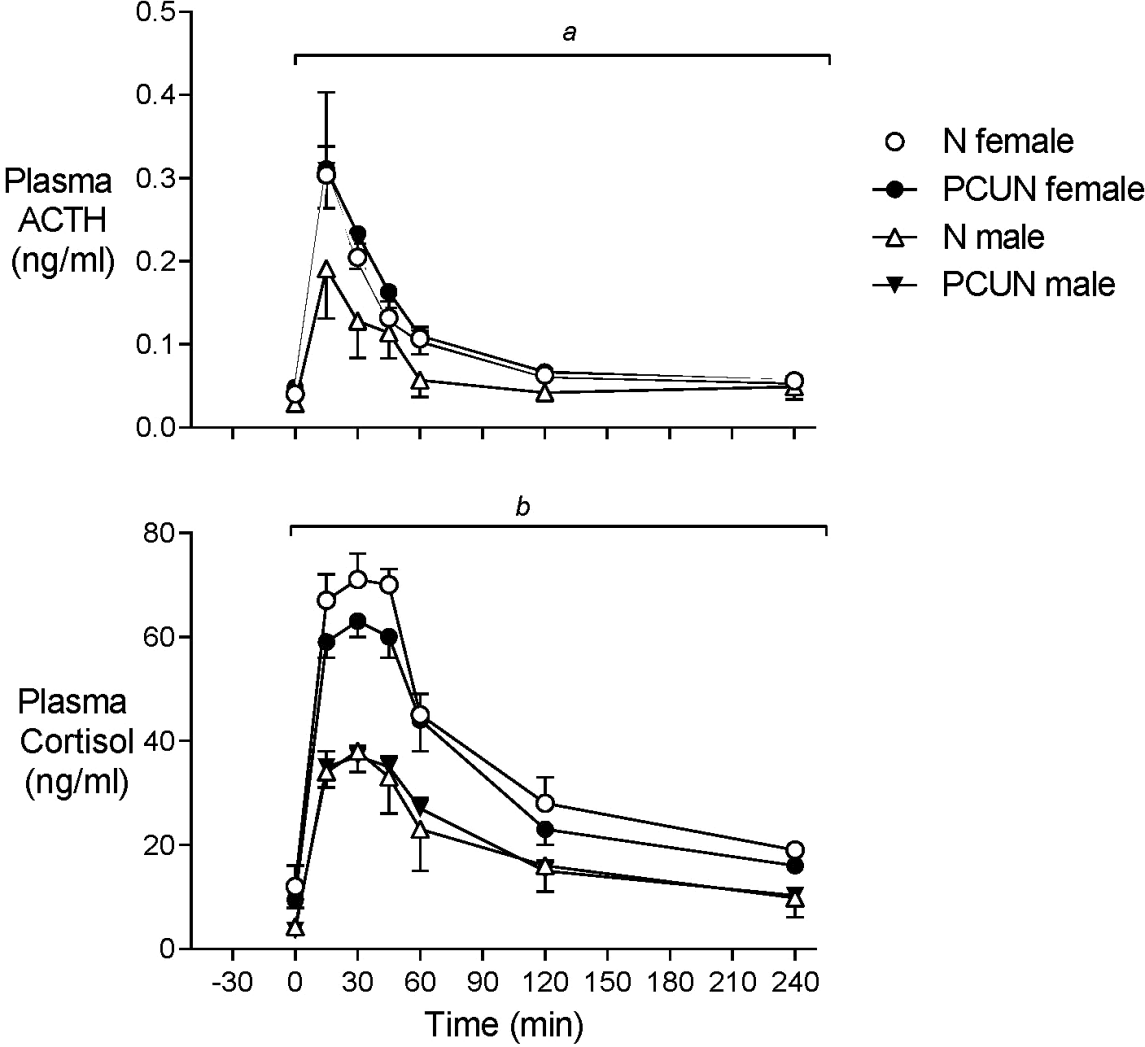
Adult plasma ACTH and cortisol responses to equimolar challenge of intravenous AVP+CRH (0.1 and 0.5 μg/kg respectively) given at time ‘0’. *^a^* treatment x time interaction in males. *^b^* sex effect (*p* < 0.0001). Values are mean ± SEM.

Plasma ACTH and cortisol concentrations before AVP+CRH challenge were not different between treatment groups, but baseline cortisol concentrations were 2-3-fold higher in females than males (**Figure 4**, N female, 12.1 ± 3.0; PCUN female, 9.5 ± 3.0 *vs*. N male, 4.3 ± 1.2; PCUN male, 3.6 ± 0.9 ng/mL, *p* < 0.05 for sex effect). Plasma ACTH AUC response (0-240 min) to AVP+CRH stimulation in females was not affected by PCUN (N, 22 ± 3 *vs*. 24 ± 3 ng/ml/min); in males, PCUN offspring had a greater AUC response than N males (22 ± 2 *vs*. 15 ± 3, ng/ml/min, *p* < 0.05, **Figure 4**). Cortisol AUC response to AVP+CRH stimulation was not affected by PCUN over 240 min (**Figure 4**); however, females had a greater cortisol AUC response than males (7861 ± 355 *vs*. 4485 ± 410 ng/ml/min, *p* < 0.0001). Plasma ACTH to cortisol AUC ratio (0-240 min) was not different between groups or between sexes (data not shown). At 240 minutes post AVP+CRH challenge plasma ACTH and cortisol concentrations were similar between groups, but cortisol concentrations remained higher in females than in males (N female, 19 ± 1; PCUN female, 16 ± 1 *vs*. N male, 9.8 ± 2.6; PCUN male, 10.3 ± 1.8 ng/mL, *p* < 0.0001).

**Figure 5:**
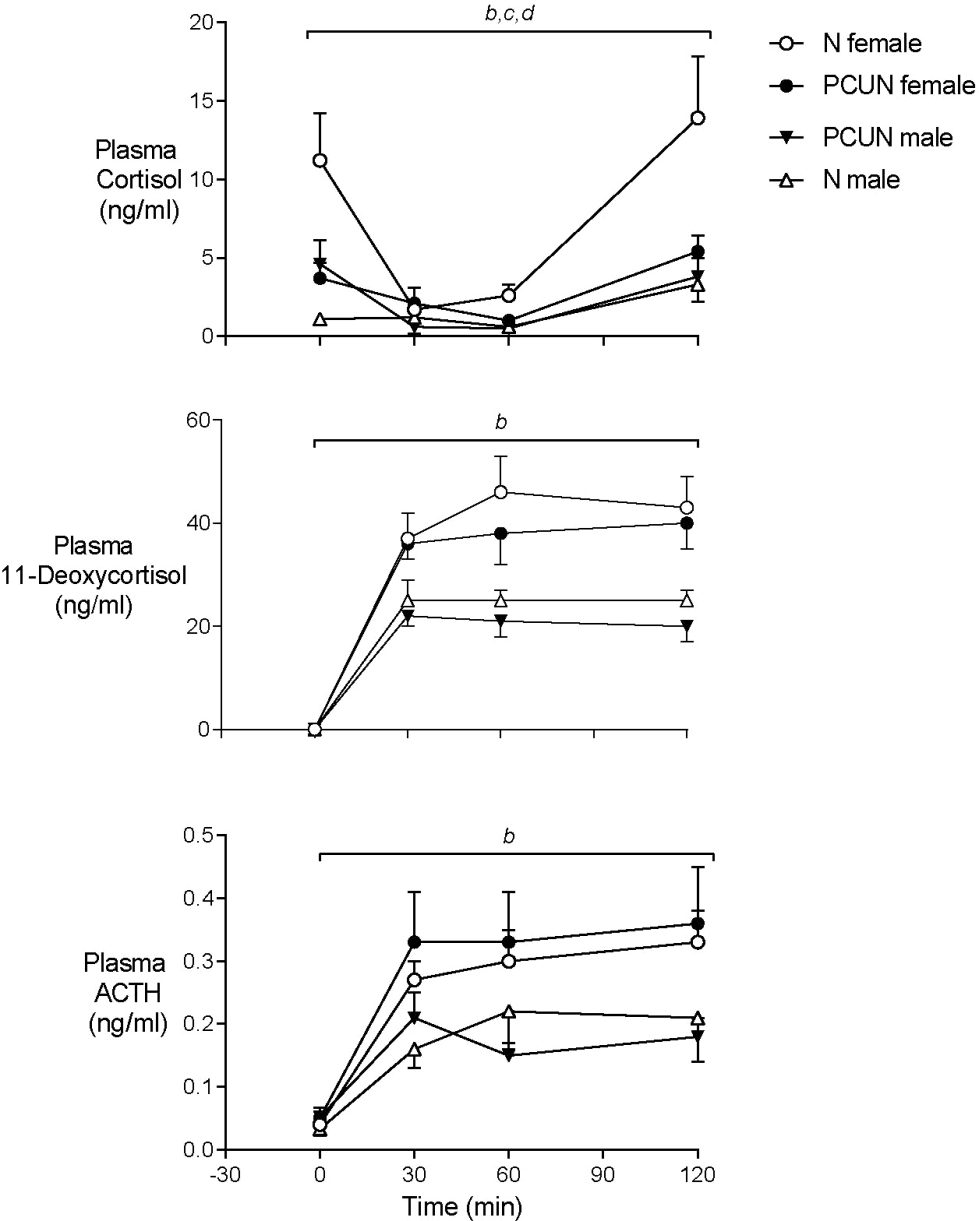
Adult plasma cortisol, 11-Deoxycortisol and ACTH responses to cortisol synthesis blockade with intravenous metyrapone (40 mg/kg) given at time ‘0’. *^b^* sex effect (*p* < 0.0001). *^c^* treatment x sex interaction (*p* < 0.05). *^d^* treatment effect in females (*p* < 0.05). Values are mean ± SEM.

Prior to metyrapone blockade of cortisol synthesis plasma concentrations of ACTH were similar in males and females of both treatment groups (**Figure 5**). Basal plasma cortisol concentrations were higher in females than males in the N group (11.2 ± 2.5 *vs*. 1.1 ± 3.5 ng/ml, **Figure 4**, *p* < 0.05) but not the PCUN group (females, 3.7 ± 1.2 *vs*. males, 4.6 ± 1.2 ng/ml). Within 30 min of intravenous injection of metyrapone, plasma cortisol had fallen significantly in all animals except N males (group*time interaction, *p* < 0.001, **Figure 4**). However, plasma concentrations of both ACTH and 11-deoxycortisol increased in both sexes of both treatment groups (*p* < 0.0001), consistent with a feedback response to blockade of cortisol synthesis, and elevations of both ACTH and 11-deoxycortisol were greater in females than in males over the 120 minutes (repeated measures, *p* < 0.05). The subsequent increase in cortisol was greater over time in N than in PCUN females (*p* < 0.05) leading to higher values at 120 min (13.9 ± 3.9 *vs*. 5.4 ± 1.0 ng/ml, *p* <0.05).

### 3.4 Adult adrenal

**Figure 6:**
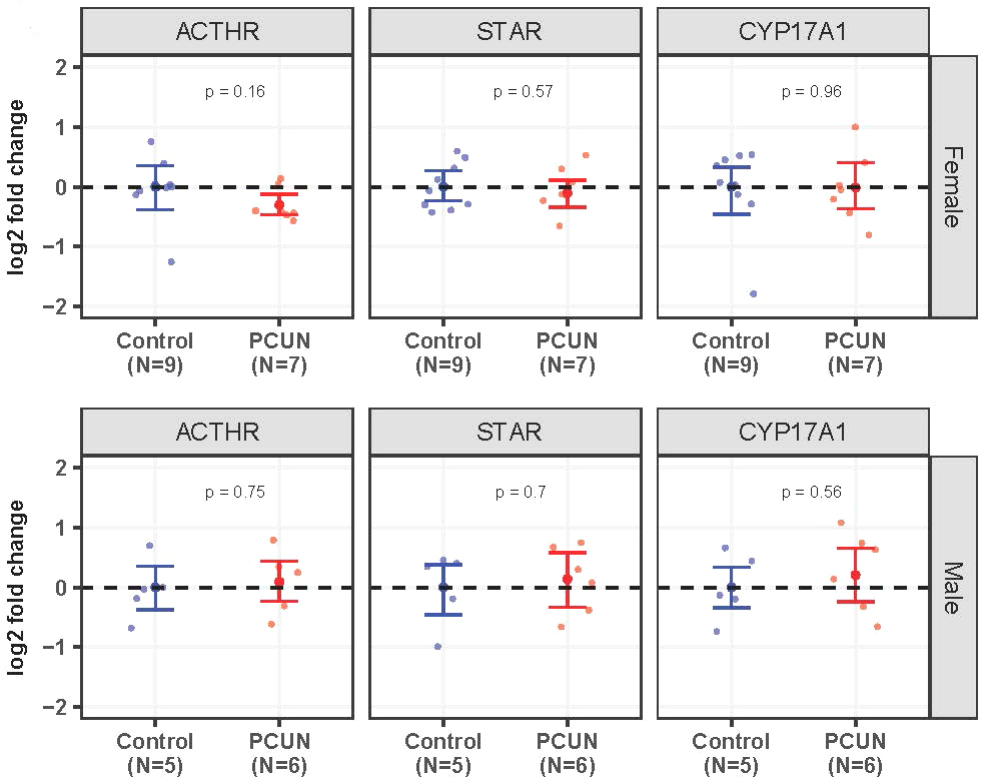
Adult adrenal mRNA expression. Log_2_ fold change of *ACTHR*, *STAR*, and *CYP17A1* mRNA expression levels in PCUN (symbol in red) in comparison to N (symbol in blue) in female **(A)** and in male **(B)**. Data are mean ± 95% confidence interval. Symbols represent individual animals. Student’s t-tests were used to assess statistical difference, with significance defined as *p* < 0.05.

Adrenal *ACTHR*, *STAR*, and *CYP17A1* mRNA expression values were not different between adult PCUN and N offspring (**Figure 6**).

## 4 Discussion

Our findings demonstrate that the effect of PCUN on the fetal adrenal steroidogenic pathway is not apparent until late in gestation, approximately 100 days after the end of the nutritional manipulation. In concert with increased fetal adrenal *STAR* and *CYP17A1* mRNA expression we also report that *IGF2* and *11β-HSD2* expression was increased in PCUN relative to N fetal adrenals at 131 days. Maturation and growth of the fetal adrenal steroidogenic pathway is influenced by a range of factors including exposure to ACTH and its precursors, and fetal growth factors like the IGFs (30). In previous studies at 131 days of gestation we have shown that PCUN leads to increased pituitary message for proopiomelanocortin (*POMC*) RNA and factors involved in post-translational processing of POMC to form functional ACTH, such as prohormone convertase-1 (2). The higher expression of *11β-HSD2* expression in PCUN adrenals may also indicate relative maturation as this enzyme is thought to be a fine-tuning modulator of cortisol secretion (31). Increased expression and activity could favor a feed forward loop favoring further development of cortisol secretion by the maturing fetal adrenal.

Previous studies in rats suggest maternal glucocorticoids mediate responses to maternal nutritional stress which in turn mediate effects on fetal HPAA development (32, 33). Studies in late-gestation sheep demonstrate that maternal exposure to synthetic glucocorticoids affects fetal HPAA development (34). We previously have reported that placental 11β-HSD2, the enzyme that inactivates cortisol to cortisone, had reduced activity at day 55, 25 days after ewes returned to a normal nutritional plane following PCUN (5). This decrease in activity was accompanied by a higher cortisol/cortisone ratio in the blood of PCUN fetuses compared to N fetuses. Glucocorticoids are known to counter-regulate mRNA expression of their own receptors (35). However, there may be crossover effects with evidence that aldosterone acting through the MR may influence both *GR* and *MR* expression in the hippocampus (36). In this study we report day 85 fetal hippocampal *GR* and *MR* mRNA expression were decreased in PCUN, relative to N fetuses, indicating that a long-term effect of PCUN had occurred. This change in fetal hippocampal *GR* and *MR* expression may indicate the start of a cascade of events that leads to earlier functional maturation of the HPAA during late gestation.

In the current studies we found no significant effect of PCUN on plasma cortisol response to AVP+CRH stimulation in female and male sheep at 3-4 years of postnatal age. Our studies at 18 months of age showed that the effect of PCUN on plasma cortisol was waning in males compared to studies at 10 months (13). Plasma ACTH responses to AVP+CRH stimulation were higher in PCUN, than in N males in the current studies. As cortisol response was not different, this suggests either adrenal ACTH receptor or post receptor mechanisms may be affected by PCUN. We do not have ACTH receptor protein or binding studies, but neither adrenal *ACTHR* mRNA nor that of *STAR* and *CYP17A* were affected by PCUN. Taken together, the AVP+CRH simulation test, cortisol synthesis blockade with metyrapone and adrenal mRNA data indicate that any effects of PCUN on cortisol secretion have largely disappeared by 3-4 years of postnatal age; approximate middle age in sheep.

Up until this investigation, studies on HPAA function in the sheep have been limited to early adulthood. Compared to the studies at 18 months, peak plasma cortisol responses to CRH+AVP had increased by 20-30% by 3-4 years of age, independent of any effect of maternal periconceptional nutrition. Studies in the human suggest that cortisol secretion increases with aging (37) so perhaps this increase in secretory activity overrides the influence of PCUN.

Although we have shown that there was a diminution of the effect of PCUN on cortisol secretory response, other studies indicate that effects of PCUN, possibly mediated by the central HPAA, do persist into mid-adulthood. For example, male PCUN offspring had increased fat mass 3-4 years of age (20) following decreased spontaneous locomotor activity at 18 months of postnatal age (38). Additional evidence comes from the observation made in animals from the current study where PCUN resulted in disturbance of hypothalamic GR and POMC epigenetic regulation, extending to both mRNA and protein expression that persisted from the late gestation fetal sheep (28) until at least 3-4 years of age (29). These persistent alterations in epigenetic and message expression are found in areas of the hypothalamus directly associated with food intake and energy balance. *GR* mRNA expression and protein were reported to be increased in male and female hypothalamus of adult PCUN sheep (29). This finding may be consistent with reports from rat studies where increased hypothalamic glucocorticoid was associated with decreased *POMC* mRNA expression (39, 40) and also obesity (41), whereas increased hypothalamic *POMC* mRNA expression following adrenalectomy in the rat led to increased hypophagic response to endotoxemia (42). Therefore, our observations of reduced locomotion and increased adiposity in adult offspring of PCUN ewes may have strong correlates in the epigenetic, mRNA and protein changes in hypothalamic GR.

The divergence in cortisol regulation between the sexes was observed at earlier postnatal ages (13) continued in the current study. Sex difference in postnatal plasma cortisol response to AVP+CRH is well reported (43–46). Similar effects of sex have been reported following the stress of tail docking lambs at 8 weeks of age (47) or other behavioral stressors (45, 48). In rats (49, 50) and humans (51) sex differences in HPAA regulation are well characterized. Sex steroids may influence this difference in HPAA regulation. For example, in the rat, acute activation of the HPAA by estrogen and inhibition by testosterone suggested as mechanisms (52). However, in human subjects a differential sex response to CRH stimulation persists during leuprolide-induced gonadal suppression (53). In our studies females were maintained in anestrus during the tests while male offspring remained uncastrated.

Strengths of this study include the longitudinal assessment from fetal life through to adulthood, with multiple fetal time-points, and the ability to manage ewe weight individually, accounting for variability in metabolism. Together with our previous studies of HPAA function in juvenile and early adult life (13), and of epigenetic modification of the glucocorticoid receptor in fetal and adult hippocampus, hypothalamus and pituitary (28, 29), these data provide a comprehensive description of maternal PCUN on the offspring HPAA. Studies in old-age sheep would have been ideal to encompass the full lifecycle but these studies already took 7 years due to breeding across years and extending them further was not viable. Similarly, additional assessments of HPAA to behavioral stress tests and investigation of immune and cardiovascular function would have been of great interest. Although the study design is relevant to the human situation, with mother and offspring kept together from conception until weaning, more mechanistic studies, such as embryo transfer (46) and/or cross fostering, are required to isolate the maternal/placental./fetal effects. From a human health perspective, the individualized nutritional management achieved a 10-15% reduction in maternal mass at mating with nutritional intake in the undernourished group approximately 80% of controls at this time (17), with no marked effect on birth size or postnatal growth (20). This is comparable with reductions in body weight that may be observed with dieting in humans and contrasts with more extreme paradigms often reported in rodent studies. The long gestation, singleton offspring and comparative fetal physiology in the ovine and human pregnancy, also are relevant for translational potential.

In conclusion, these studies further clarified the effect of PCUN on fetal HPAA development in late gestation and provide further evidence that maternal nutrition around the period of conception has important effects that last into middle-age offspring. We have shown in related studies that hypothalamic effects on central HPAA elements have remained present in 3–4-year-old sheep (29) and may be consistent with altered behavioral (38) and body composition phenotypes (20). However, the regulation of peripheral circulating cortisol concentrations in response to the corticotropic stimulation tests used, has normalized, in contrast with the findings in the same animals at earlier ages (13). A life stage-specific HPAA regulation by PCUN may represent an appropriate ‘trade off’ adaptation (54) given the central role the axis has on functional maturation in late gestation, in preparation for survival from birth and then later in life, on regulation of energy balance and fat mass. Effects of changed HPAA regulation with age on other systems, e.g., the immune, have yet to be determined within this PCUN paradigm. In addition, we confirm that changes in HPAA regulation with postnatal age interact with sex of the offspring. The summed information from our own and other studies, from late gestation to 3–4-year-old adult sheep, clearly show that related endocrine and metabolic axes may be affected differently at different times of life by PCUN and therefore isolated snap-shot observations at a specific age may not be representative of the complex biology resulting from restricted maternal nutrition during the periconceptional period.

## Data availability statement

The datasets presented in this study can be found in online repositories. The names of the repository/repositories and accession number(s) can be found below: https://figshare.com/, 4148055972811865db05; https://figshare.com/, 83a677c9ee88b2989ad4.

## Ethics statement

The animal study was reviewed and approved by the University of Auckland Animal Ethics Committee.

## Author contributions

MO, AJ, KC, JH, and FB conceptualized the hypothesis and designed the experiment. MO and AJ performed the animal studies. KC and HP designed the molecular approaches used and performed that analysis. ET supervised the ACTH RIAs, designed the LQ tandem mass-spectroscopy approach and performed the analysis. MO and HP performed the statistical analysis. MO led the manuscript drafting and all authors contributed. All authors contributed to the article and approved the submitted version.
